# Small renal cell carcinoma accompanied by extensive inferior vena cava tumor thrombus diagnosed by percutaneous transvenous biopsy

**DOI:** 10.1002/iju5.12662

**Published:** 2023-11-01

**Authors:** Hikaru Tsukada, Nozomi Hayakawa, Koichiro Aida, Shinji Wada, Tsuyoshi Morimoto, Masatomo Doi, Hidefumi Mimura, Junki Koike, Eiji Kikuchi

**Affiliations:** ^1^ Department of Urology St. Marianna University School of Medicine Kawasaki Japan; ^2^ Department of Diagnostic and Interventional Radiology St. Marianna University School of Medicine Kawasaki Japan; ^3^ Department of Diagnostic Pathology St. Marianna University School of Medicine Kawasaki Japan

**Keywords:** differential diagnosis, inferior vena cava, small renal cell carcinoma, transvenous biopsy, tumor thrombus

## Abstract

**Introduction:**

Up to 10% of patients with renal cell carcinoma present with tumor thrombus in the inferior vena cava. We report that a case of small renal cell carcinoma with tumor thrombus extending above the diaphragm for which transvenous biopsy was performed for diagnosis.

**Case presentation:**

A 79‐year‐old man performed computed tomography to evaluate hepatic dysfunction, which revealed intravenous tumor extending above the diaphragm and a 15‐mm‐sized exophytic tumor in right kidney. Imaging suggested that the renal tumor was renal cell carcinoma. As this tumor was small and exophytic, confirmation of the intravenous tumor being tumor thrombus associated with renal cell carcinoma was difficult. We simultaneously performed transvenous biopsy on the intravenous tumor and percutaneous biopsy on the renal tumor for obtaining histologic diagnoses. The final diagnosis was small renal cell carcinoma accompanied by tumor thrombus above the diaphragm.

**Conclusion:**

Transvenous biopsy may be useful for the definitive diagnosis of inferior vena cava‐tumor thrombus in cases of small renal cell carcinoma.

**Abbreviations & Acronyms**
CT: computed tomography
IVC: inferior vena cava
MRI: magnetic resonance imaging
RCC: renal cell carcinoma
TT: tumor thrombus
US: ultrasonography

**Keynote message**

Small renal cell carcinoma is rarely accompanied with inferior vena cava tumor thrombus expanding above the diaphragm. In such cases, especially if continuity between the renal cell carcinoma and tumor thrombus cannot be confirmed, we sometimes need to make pathological diagnosis. Herein, we describe a case of the exophytic small cell carcinoma with inferior vena cava tumor thrombus expanding above the diaphragm diagnosed by transvenous tumor thrombus biopsy.

## Introduction

Some RCC patients have TT in the IVC at the time of initial presentation, which rarely can reach the cardiac chambers.^1^, ^2^, ^3^, ^4^ It is generally recognized that the primary renal tumor size is relatively large in patients with TT in IVC and especially the right atrium, because it takes time for renal tumor to invade the venous system with extension into the renal vein, IVC, and cardiac chambers. We report that a case of small RCC (1.5 × 1.3 cm) with venous thrombus reaching the right atrium, which was histologically confirmed by percutaneous transvenous biopsy.

## Case presentation

**Fig. 1:**
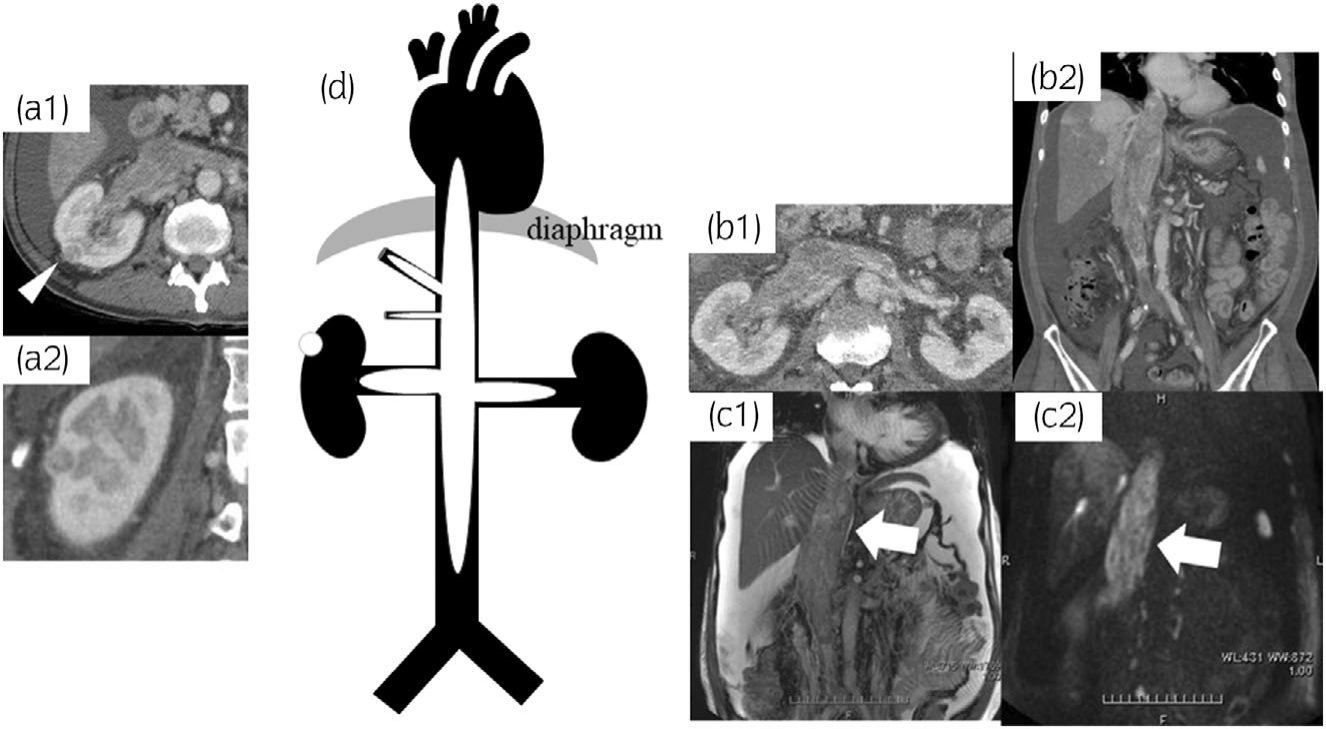
Imaging findings. Contrast‐enhanced CT showing exophytic right 15 x 13 mm renal tumor, axial view (arrowhead, tumor) (a‐1); coronal view (a‐2). Contrast‐enhanced CT showing an intravenous tumor extending from the right atrium to the L4 vertebral level and into the bilateral renal veins with right‐sided predominance, axial view (b‐1); coronal view (b‐2). Coronal view of MRI showing an intravenous tumor (arrow) with heterogeneous high intensity on T2‐weighted imaging (c‐1) and with hyperintensity on diffusion‐weighted imaging (c‐2). Schematic view of small renal mass and the extent of intravenous tumor (d).

**Fig. 2:**
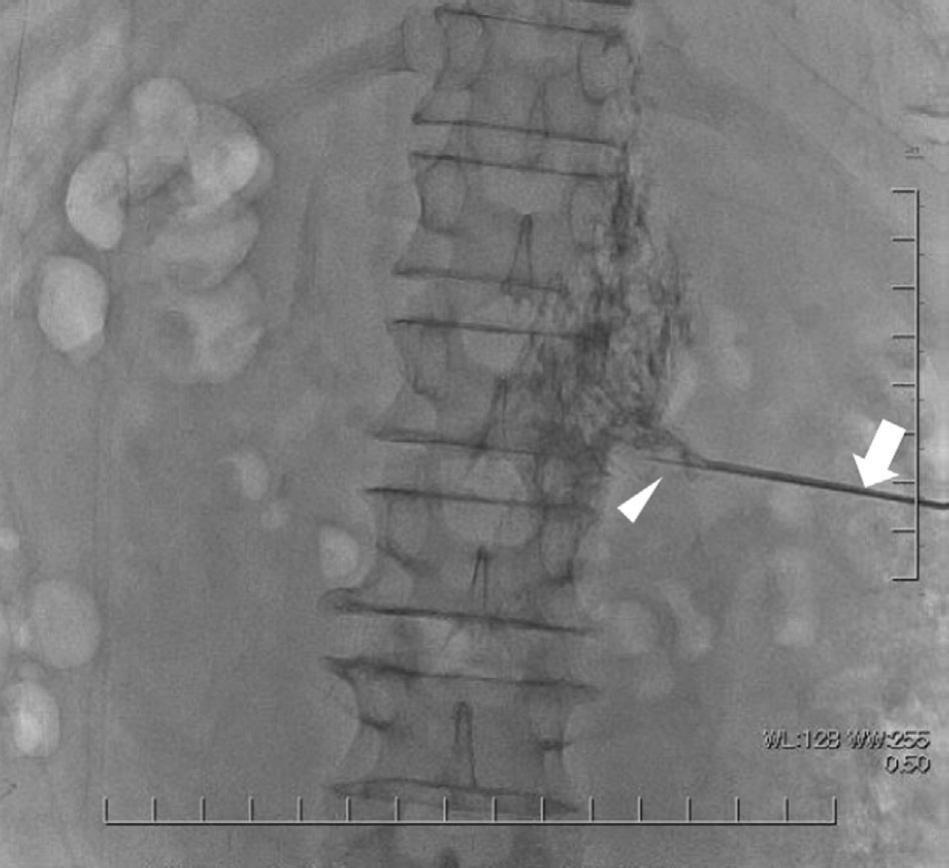
Fluoroscopic X‐ray image at the time of intravenous tumor (arrowhead: 22 G percutaneous transhepatic cholangiography needle; arrow: 17 G hollow needle).

**Fig. 3:**
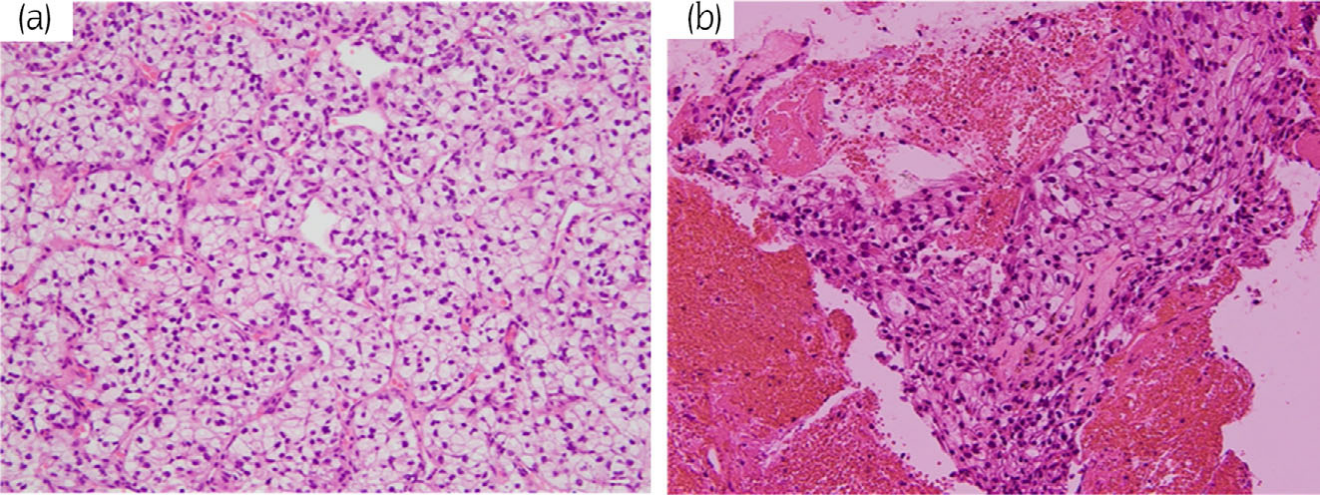
Pathological findings (×20). The specimens of renal tumor revealed that tumor cells had abundant pale cytoplasm in hematoxylin and eosin (HE) staining (a) and the morphologic feature of intravenous tumor specimens was similar to renal tumor (b).

A 79‐year‐old man visited our institution because of an extensive IVC tumor and a small right renal tumor. He had had edema in bilateral lower extremities for 2 months and also felt abdominal distension from 3 weeks before. He underwent CT and blood test to investigate the cause of his symptoms at the hospital where his type 2 diabetes mellitus was being treated. CT showed an extensive intravenous tumor in the IVC causing hepatic ischemia and ascites and concurrently indicated small right renal tumor. He visited our department due to suspected IVC‐TT associated with RCC. His blood test revealed abnormal values of Hb (11.1 g/dL), AST (191 U/L), ALT (446 U/L), and CRP (4.73 mg/dL), but other laboratory data were within normal range. Dynamic CT, abdominal MRI, and US were performed (Fig. 1). Dynamic CT revealed a renal mass of 15 mm in size on the right lateral aspect in corticomedullary phase, suggesting RCC. The intravenous tumor was enhanced heterogeneously, expanding the lumen and extending from the right atrium to the L4 vertebral level and also into the bilateral renal veins with right‐sided predominance. This intravenous tumor was suspected to have arisen from the smooth muscle of the IVC wall, such as leiomyosarcoma, rather than TT associated with RCC because the renal tumor was too small for extensive IVC thrombus and the direct invasion of the small renal tumor into the renal vein could not be detected by MRI and US. To confirm the histological diagnosis, we performed biopsies of both the intravenous and renal tumors. Eight days after the patient's first visit to our hospital, he simultaneously underwent an ultrasound‐guided biopsy for the small renal tumor and a percutaneous transvenous biopsy for the intravenous tumor. The procedure of the percutaneous transvenous technique is summarized in Table 1 and Fig. 2. Based on pathological findings and no finding of other metastatic lesions, the final diagnosis was clear cell RCC with IVC‐TT (Fig. 3).

**Table 1:** Percutaneous transvenous biopsy technique for intravenous tumor

Step 1	Under local anesthesia, the patient was placed in the left lateral decubitus position. All procedures were performed under X‐ray fluoroscopy.
Step 2	A 22 Gauge (G) percutaneous transhepatic cholangiography (PTC) needle was inserted under ultrasound guidance into the right renal vein through the right kidney.
Step 3	After removing the needle stylet, the intravenous tumor was detected by injecting a small amount of contrast material.
Step 4	A 17 G hollow needle was inserted into the renal vein parallel to the 22 G PTC needle (Fig. 2). The 22 G PTC needle was then withdrawn.
Step 5	Specimens were taken from five different regions of intravenous tumor with a 18 G biopsy needle passed through the 17 G hollow needle while confirming its location with contrast under fluoroscopy.

The patient's general condition rapidly deteriorated from KPS 60 at his initial visit to 40 in just 3 weeks. Therefore, he and his family chose supportive care at their home, and declined surgery and systemic therapy such as immune checkpoint inhibitors.

## Discussion

Small renal masses (≤4 cm) are believed to be of indolent pathology. Active surveillance may be a suitable option for small renal tumor in elderly and comorbid patients. However, in rare occasions, small RCC has metastatic potential. There have been some previous reports of IVC‐TT in small RCC.^1^, ^2^, ^3^, ^5^, ^6^, ^7^, ^8^ A retrospective cohort study of 1192 RCC with TT patients at 13 European institutions indicated that of 63 patients who had IVC‐TT above the diaphragm, no patients had a primary RCC size of less than 4 cm.^1^ The median renal tumor diameter ranged from 8 to 11 cm in patients with IVC‐TT in most previous reports.^2^, ^3^, ^5^, ^6^ On the contrary, Haddad *et al*. reviewed 166 cases of RCC with suprahepatic venous thrombus and reported that the minimum primary tumor diameter was 22 mm.^6^ To the best of our knowledge, the present primary tumor diameter of 15 mm is the smallest reported in patients with IVC‐TT above the diaphragm.

In the present case, the primary RCC was small and the direct invasion of the tumor from renal tumor to renal vein could not be detected on imaging, making the definitive diagnosis difficult. The first impression was tumor arising from the smooth muscle of the IVC wall on imaging. Subsequently, we planned to perform biopsy for the IVC tumor transvenously to confirm the histological diagnosis. Transvenous biopsy for malignancy is an established technique, which was first reported by Robins and Bookstein in 1972,^9^ but underused as an alternative to open or percutaneous biopsy for the diagnosis of malignancy. However, only small case series for the technique have been described the safety and accuracy of transvenous biopsy for malignancy.^10^, ^11^ To the best of our knowledge, only three case reports utilizing the technique in RCC patients with IVC‐TT have been published previously.^11^, ^12^, ^13^ These previous studies reported that a definitive histopathologic diagnosis could be obtained in all cases, and no severe intra‐ or postprocedural complications were observed. Although the histological diagnosis of TT is rarely necessary in RCC cases, transvenous biopsy may be a valuable diagnostic tool to avoid tumor seeding from intravenous TT.

In this case, a small and extravasated RCC was accompanied by extensive and aggressive TT. Although it is only speculation since no autopsy was performed, we suspect that the TT in this case was a metastatic lesion and not a direct invasion of RCC. Our hypothesis is supported by previous studies that reported that metachronous TT in IVC occurred after radical nephrectomy for localized RCC. Malignant potential was not always concordant between primary tumor and intravenous recurrence lesion; for example, in one case, the IVC‐TT below the diaphragm was detected 6 months after radical nephrectomy for T1a RCC.^14^ Furthermore, Minervini *et al*. hypothesized that metachronous TT in the IVC after radical nephrectomy was caused by thrombus that originated from microscopic involvement of the IVC wall, which was already present at the time of the operation, not in continuity with the primary RCC.^15^ In this case, we believe the tumor cells that flowed into the venous system from the primary tumor adhered to the IVC wall and multiplied rapidly regardless of growth of the primary tumor.

## Conclusion

We encountered a case of a small size of RCC accompanied by an extensive and aggressive IVC‐TT above the diaphragm, which was diagnosed by percutaneous transvenous biopsy.

## Author contributions

Hikaru Tsukada: Conceptualization; investigation; visualization; writing – original draft. Nozomi Hayakawa: Conceptualization; project administration; resources; writing – review and editing. Koichiro Aida: Resources. Shinji Wada: Resources; visualization; writing – review and editing. Tsuyoshi Morimoto: Resources; writing – review and editing. Masatomo Doi: Resources; writing – review and editing. Hidefumi Mimura: Supervision; writing – review and editing. Junki Koike: Resources; supervision. Eiji Kikuchi: Project administration; supervision; writing – review and editing.

## Conflict of interest

The authors declare no conflict of interest associated with this manuscript.

## Approval of the research protocol by an Institutional Reviewer Board

Not applicable.

## Informed consent

Written informed consent of the patient's family has been obtained, because the patient is deceased.

## Registry and the Registration No. of the study/trial

Not applicable.
